# The effect of BLA GABA(A) receptors in anxiolytic-like effect and aversive memory deficit induced by ACPA

**DOI:** 10.17179/excli2015-201

**Published:** 2015-05-11

**Authors:** Katayoon Kangarlu-Haghighi, Shahrbanoo Oryan, Mohammad Nasehi, Mohammad-Reza Zarrindast

**Affiliations:** 1 Department of Biology, Science and Research Branch, Islamic Azad University, Tehran, Iran; 2 Cognitive and Neuroscience Research Center (CNRC), Medical Genomics Research Center and School of Advanced Sciences in Medicine, Islamic Azad University, Tehran Medical Sciences Branch, Tehran, Iran; 3 Department of Pharmacology School of Medicine, Tehran University of Medical Sciences, Tehran, Iran; 4 Iranian National Center for Addiction Studies, Tehran University of Medical Sciences, Tehran, Iran; 5 School of Cognitive Sciences, Institute for Research in Fundamental Sciences (IPM), Tehran, Iran

**Keywords:** ACPA, GABA, anxiety, memory, amygdala

## Abstract

The roles of GABAergic receptors of the Basolateral amygdala (BLA) in the cannabinoid CB1 receptor agonist (arachydonilcyclopropylamide; ACPA)-induced anxiolytic-like effect and aversive memory deficit in adult male mice were examined in elevated plus-maze task. Results showed that pre-test intra-peritoneal injection of ACPA induced anxiolytic-like effect (at dose of 0.05 mg/kg) and aversive memory deficit (at doses of 0.025 and 0.05 mg/kg). The results revealed that Pre-test intra-BLA infusion of muscimol (GABA_A_ receptor agonist; at doses of 0.1 and 0.2 µg/mouse) or bicuculline (GABA_A_ receptor antagonist; at all doses) impaired and did not alter aversive memory, respectively. All previous GABA agents did not have any effects on anxiety-like behaviors. Interestingly, pretreatment with a sub-threshold dose of muscimol (0.025 µg/mouse) and bicuculline (0.025 µg/mouse) did not alter anxiolytic-like behaviors induced by ACPA, while both drugs restored ACPA-induced amnesia. Moreover, muscimol or bicuculline increased and decreased ACPA-induced locomotor activity, respectively. Finally the data may indicate that BLA GABA_A _receptors have critical and different roles in anxiolytic-like effect, aversive memory deficit and locomotor activity induced by ACPA.

## Introduction

Cannabis sativa (Marijuana) is commonly used all over the world. The plant extract composes of almost 70 cannabinoid compounds (Burns, 2006[11]). Various researches have shown that the administration of marijuana affects various cognitive and non-cognitive behaviors including impairment of spatial (Uchida et al., 2012[71]; Wise et al., 2009[79]) and non-spatial learning and memory, anxiety-like behaviors, mood, locomotor, and euphoria experience both in animal models and human subjects (Burgdorf et al., 2011[10]; Kilmer et al., 2011[34]; King et al., 2002[35]; Pacula, 2011[58]). Some reports revealed that the endogenous cannabinoid system is critically linked to the extinction of aversive memories (Marsicano et al., 2002[44]). Endocannabinoids are thought to be retrograde messengers released by neurons to modulate release of neurotransmitters (Kreitzer and Regehr, 2001[37]; Nicolle et al., 2001[55]; Ohno-Shosaku et al., 2001[56]; Wilson and Mogil, 2001[78]). Three main cannabinoid receptors have been identified so far as CB1, CB2, and CB3 (non-CB1 and -CB2), which are engaged in cannabinoids' functions (Mackiewicz et al., 2006[41]; Ryberg et al., 2008[67]). CB1 receptors are plentifully expressed in the central nervous system regions such as the hippocampus, amygdala, cerebellum and cortex (Davies et al., 2002[18]; Pertwee and Ross, 2002[61]; Wilson et al., 2002[77]). CB2 receptors mostly are expressed peripherally rather than in brain tissues. It is believed that neuropsychological functions of endocannabinoids are related to CB1 receptors. CB1 is mainly expressed in the amygdala (Katona et al., 2001[33]; McDonald and Mascagni, 2001[45]), an essential part and component of the limbic circuitry. The amygdala is an integral part in controlling the emotional behavior such as conditioned fear, anxiety (McKernan and Shinnick-Gallagher, 1997[47]), and pain perception (Gauriau and Bernard, 2002[23]; Paulson et al., 2002[59]). 

Amygdala nuclei mainly are classified into the three groups:

1. the deep or basolateral group, which includes the lateral nucleus, the basal nucleus, and accessory basal nucleus used as auxiliary and helping nucleus;
2. the superficial or cortical-like group, which consists of cortical nuclei and nucleus of the lateral olfactory tract; and
3. the centromedial group composed of the medial and central nuclei (Faber and Sah, 2003[21]; Sah and Lopez De Armentia, 2003[68]).

It has been proven that CB1 cannabinoid receptors are expressed at high levels in the BLA amygdala nuclei (Herring et al., 2003[29]; Marsicano et al., 2002[44]). Expression of the CB1 protein is limited to a definite and noticeable subpopulation of GABAergic interneurons corresponding to large cholecystokinin-positive cells (Jazi et al., 2009[31]). In-depth and comprehensive analysis has shown that CB1 receptors exist presynaptically on cholecystokinin-positive axon terminals, which establish symmetrical GABAergic synapses with their postsynaptic targets (Azad et al., 2003[5]; Katona et al., 2001[33]). In the mammals brain, γ-aminobutyric acid (GABA) is the most abundant inhibitory neurotransmitter (Nicoll et al., 1990[54]), working by means of different receptor types: the ionotropic GABA_A_ and GABA_C_ receptors (both of which activate Cl^−^ currents) and the metabotropic GABA_B_ receptor (G protein coupled receptor). The present study, in accordance with the above mentioned data, was designed to examine the role of BLA GABAergic (GABA_A_ receptors) system on the ACPA (selective CB1 cannabinoid receptor agonist), which causes emotional amnesia in the EPM test-retest protocol in mice. 

## Materials and Methods

### Animals

Male albino NMRI (Institute Pasture, Iran) mice weighting 27-30 g (9-10 week old), were applied. The animals were kept under a 12/12-h light-dark cycle, with light beginning at 0700 h and at a controlled temperature (22 ± 2 c). They had free access to food and water. The animals were housed 10 per cage (45 cm × 30 cm × 15 cm). Eight animals were used in each experiment. Each animal was only used once. All procedures in this investigation are in accordance with the guide for the Care and Use of Laboratory Animals as adopted by the Ethics Committee of Faculty of Science, Tehran University (357: November 2000).

### Drugs 

Ketamine and xylazine (Alfasan Chemical Co., Woerden, Holland) were applied for animal anesthesia. Muscimol (GABA_A _receptor agonist), bicuculline (GABA_A_ receptor antagonist) and ACPA (CB1 cannabinoid receptor agonist) have been used in this study that purchased from Tocris, Bristol, UK). All drugs were dissolved in sterile 0.9 % saline, just before the experiment, except for bicuculline. Bicuculline was dissolved in 1 drop of glacial acetic acid using a Hamilton microsyringe, then made up to a volume of 5 ml with sterile 0.9 % saline and then diluted to the required volume. Muscimol and bicuculline were administered into the BLA of amygdala at volume of 0.6 µl/ mouse (0.3 µl/site). The control animals received saline or vehicle. The timing of drugs administration was defined as per our pilot results and the previous studies (Chegini et al., 2014[15]; Yousefi et al., 2013[81]).

### Surgical procedures and microinjections

All surgical procedures were organized under ketamine-xylazine (100 mg/kg ketamine-5 mg/kg xylazine) anesthesia. Cannulae were implanted with bilateral 27-gauge stainless steel cannulas into either the BLA of amygdala. Drugs were injected into the amygdala (coordinates from bregma as follows: AP = -0.7 mm, ML = ± 2.7, DV = 3. 8 (Paxinos and Franklin, 2001[60]). Skull cap was made from dental acrylic. Finally, stainless steel wires were inserted with plastic caps into each cannula to prevent any debris from entering the brain and to maintain patency of the hollow metal cylinder. The injecting needle extended 1 mm beyond the tip of the cannulas, reflecting the ultimate desired depth of the apparatus and the actual depth the infusion needle reached during antagonist or buffer infusion. Following surgery all animals were allowed 1 week to recover from surgery and get cleared from anesthetics effect. For drug infusion, animals were gently restrained in hand and the stylets were removed from the guide cannulae and replaced by 27-gauge injection needles. Each injection unit was connected by polyethylene tubing to a 1 µl Hamilton syringe. The forward movement of a small air bubble inside the polyethylene tubing interposed among the upper end of needle and the microsyringe was taken as evidence of drug flow. The injection needles were left in place for an additional 60 s to allow diffusion after which the stylets were reinserted into the guide cannulae (Ebrahimi-ghiri et al., 2012[20]; Nasehi et al., 2012[52]; Zarrindast et al., 2011[84]). 

### Apparatus and behavioral testing

We applied a wooden elevated plus-maze (EPM) apparatus set up 50 cm above the floor which included two oppositely positioned open-arms (50 × 10 cm) and two enclosed arms (50 × 10 × 40 cm), surrounded by1cm high Plexiglas ledge so that to prevent falls. The junction area of the four arms (central platform) measured 10 × 10 cm (Carobrez and Bertoglio, 2005[13]; Zarrindast et al., 2010[83], 2011[85]). The EPM test is used to assess anxiety and memory processes in rodent models of CNS disorders. 

Findings demonstrate that aversive learning and memory may be studied at the same time as anxiety in rodents exposed to the EPM test/retest. Animals retested in the EPM avoid exploring the open spaces, displaying a clear enclosed arm preference with a low percentage of entries and time spent in the open arms relative to their respective level on testing. The aversive and fear-inductor nature of the open arms represents a useful tool for the study of aversively motivated learning processes in the EPM. For example, learning and memory have been studied in the EPM through avoidance to open-arms in the retest session. The different analysis indicated that this response of further avoidance to open-arms is gradually acquired throughout testing, and is thought to reflect the retrieval of the aversive memory related to the initial EPM exploration (Chegini et al., 2014[15]; Valizadegan et al., 2013[73]).

Mice were left undisturbed to the testing room 1 hour prior to the test so that to adapt to the testing environment. The mice were individually placed in the center of the maze facing a closed arm and allowed 5 min of free exploration. Experiments were under a low light (40-lux), during the day phase, between 9:00 and 14:00 h. During this 5 min, the percentage of open arm time and open arm entries were calculated as follows:

- %OAT (the ratio of time spent in the open arms to total time spent in any arms×100);
- %OAE (the ratio of entries into open arms to total entries×100) and CAE (close arms entries as a relative pure index of locomotor activity).

These behaviors were recorded by a video camera while a monitor and a computer-recording system were installed in an adjacent room. Raw data were used to manually calculate these behaviors. Experiments were performed by someone blind to doses of drugs and statistical results. 

### Experimental design

#### Experiment 1: Effect of pretest microinjections of muscimol and bicuculline on open-arm exploratory-like behaviors

To substantiate that the microinjection of drugs into BLA involves in anxiety, the drug infusion took place before EPM testing. In the present experiment nine groups of animals received saline (0.6 µl/mouse, 3 groups), vehicle (0.6 µl/mouse, 3 groups), muscimol (0.025, 0.05, 0.1, and 0.2 µg/ mouse) or bicuculline (0.025, 0.05, 0.1, 0.2 and 0.4 µg/mouse), 5 min. before testing. In order to investigate possible after-effect intra-BLA drugs effects on aversive learning during test day to aversive memory in retest day, treated groups were retested in the EPM 24 h later un-drugged. 

#### Experiment 2: Effect of pretest microinjections of ACPA on open-arm exploratory-like behaviors 

To provide information on the impact of ACPA on anxiety, the drug infusion happened before EPM testing. In this experiment 4 groups of animals received saline (10 ml/kg, i.p.) and ACPA (0.0125, 0.025, and 0.05 mg/kg, i.p.) 15 minutes before testing. To inquire possible carry-over drug effects on aversive learning, treated groups were retested in the EPM 24 h later un-drugged. 

#### Experiment 3: Effect of pretest microinjections of muscimol and bicuculline on open-arm exploratory-like behaviors induced by ACPA

To supply evidence that possible interaction of GABA_A _ BLA receptors with exploratory-like behaviors induced by ACPA, the drugs infusion were made before EPM testing for anxiety-like behavior assessment. In these experiments the animals received saline (0.6 µl/mouse, 4 groups), sub-threshold dose of muscimol (0.025 µg/mouse, 4 groups) and bicuculline (0.025 µg/mouse, 4 groups) intra-BLA, 5 min before testing. Furthermore, these animals also received saline (10 ml/kg, i.p.) and sub-threshold and effective doses of ACPA (0.0125, 0.025 and, 0.05 mg/kg, i.p.) 15 min before testing. In order to look into the possible side-effects of intra-BLA drugs on aversive learning, treated groups were retested in the EPM 24 h later un-drugged. 

### Verification of cannulae placements

After the completion of the experimental sessions, each animal was eliminated with an overdose of chloroform. Animals received intra-BLA injection of ink (0.3 l/side; 1 % aquatic methylene blue solution). The brains were then removed and fixed in a 10 % formalin solution for 10 days before sectioning. Sections were analyzed to find out the location of the cannulae aimed for bilateral BLA. The cannulae placements were checked by using the atlas of Paxinos and Franklin (2001[60]). Data which were obtained from animals with injection into the specified sites, outside these regions were not taken into the consideration for the analysis. 

### Statistical analysis

Given the normality of distribution and the homogeneity of variance, the results were statistically evaluated using the repeated measure and two-way analysis of variance (ANOVA), in which mean ± S.E.M was employed for the comparison of outcomes between experimental groups and their corresponding controls. Where *F*-value was significant, one-way analysis of variance (ANOVA) and post-hoc analysis (Tukey's test) were performed. Differences with *P* < 0.05 between groups were considered statistically significant.

## Results

### Histology

For the statistical analyses, we included the data only from animals with correct cannulae implants (320 animals). 27 animals with incorrectly positioned cannulae tips were excluded.

### Effect of pretest microinjections of muscimol into BLA on open-arm exploratory-like behaviors

**Figure 1:**
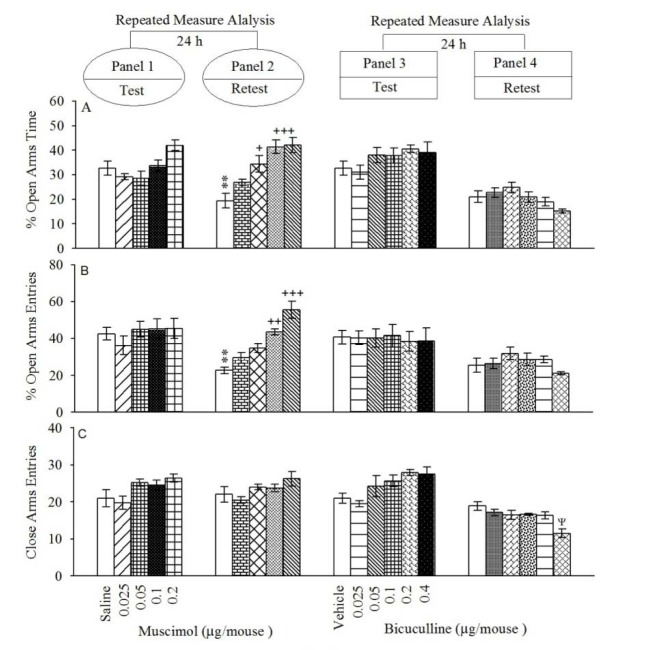
Open-arms exploratory behavior following pretest microinjections of muscimol (Panels 1 and 2) and bicuculline (Panels 3 and 4) into BLA. After 24 h, all groups were retested in the EPM un-drugged. %OAT (A); %OAE (B) and CAE (C). Values are expressed as mean±S.E.M (n=8 in each group). **P < 0.05 different from respective saline group in the panel 1. +P < 0.05, ++P < 0.01 and +++P < 0.001 different from control saline group in Panel 2. ψ < 0.05 different from the saline group in the Panel 4.

Repeated measure and post-hoc analysis demonstrated that intra-BLA injection of muscimol at dose 0.1 and 0.2 µg/mouse increased %OAT (Figure 1(Fig. 1); Panel 2A) and %CAE (Figure 1(Fig. 1); Panel 2B) but not CAE (Figure 1(Fig. 1); Panel 2C) in the retest day, while these interventions did not alter all behaviors in the test day (Figure 1(Fig. 1); Panels 1A, 1B and 1C). In conclusion, the data uncovered that muscimol did not induce any effect on anxiety behaviors, while impaired aversive memory acquisition. All the experimental repeated measure results are summarized in the Table 1(Tab. 1).

### Effect of pretest microinjections of bicuculline into BLA on open-arm exploratory-like behaviors

Repeated measure and post-hoc analysis demonstrated that intra-BLA injection of bicuculline at dose 0.4 µg/mouse decreased CAE (Figure 1(Fig. 1); Panel 4C) but did not alter %OAT (Figure 1(Fig. 1); Panel 4A) and %CAE (Figure 1(Fig. 1); Panel 4B) in the retest day. The interventions did not alter any behaviors in the test day (Figure 1(Fig. 1); Panels 3A, 3B and 3C). In conclusion, the data showed that bicuculline did not induce any effect on anxiety behaviors and aversive memory acquired. All the experimental repeated measure results are summarized in the Table 1(Tab. 1).

### Effect of pretest intraperitoneal injections of ACPA on open-arm exploratory-like behaviors

**Figure 2:**
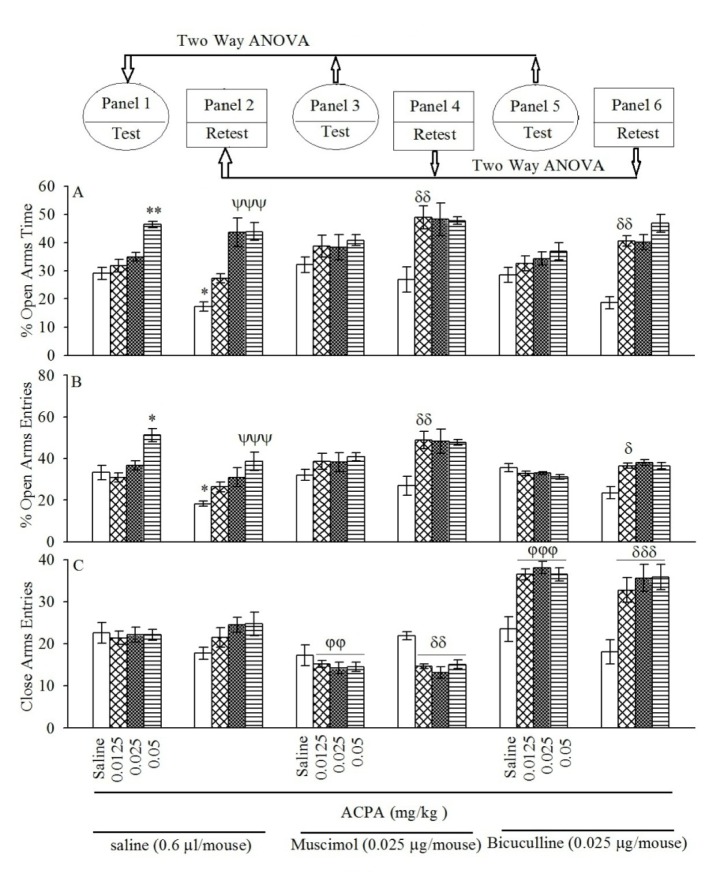
Panels 3 and 4 for muscimol and Panels 5 and 6 for bicuculline show the effect of intra-BLA pre-testing injection of sub-threshold dose muscimol and bicuculline on open-arms exploratory-like behavior induced by both sub-threshold and effective doses of ACPA. After 24 h, all groups were retested in the EPM un-drugged which showed by %OAT (A), %OAE (B) and CAE (C). Values are expressed as mean±S.E.M (n = 8 in each group). *P < 0.05 and **P < 0.01 different from saline group in the Panel 1. ψψψP < 0.001 different from saline group in Panel 2. For panels 3 φφ < 0.01 and φφφ < 0.001 are compared to respective group in the Panel 1, while δ < 0.05, δδ < 0.01 and δδδ < 0.001 are compared to the respective group in the Panel 2.

Repeated measure and post-hoc showed that intraperitoneal injection of ACPA at the highest dose (0.05 mg/kg) increased %OAT (Figure 2(Fig. 2); Panel 1A) and %CAE (Figure 2(Fig. 2); Panel 1B) but not CAE (Figure 2(Fig. 2); Panel 1C) in the test day. The same interventions in the retest day showed that ACPA increased %OAT (Figure 2(Fig. 2); panel 2A at doses 0.025 and 0.05 mg/kg) and %CAE (Figure 2(Fig. 2); Panel 2B at dose 0.05 mg/kg) but not CAE (Figure 2(Fig. 2); Panel 2C). In conclusion, the data demonstrated that ACPA induced anxiolytic-like effect and impaired aversive memory acquisition. All the experimental repeated measure results are summarized in the Table 2(Tab. 2). 

### Effect of pretest microinjections of muscimol and bicuculline on open-arm exploratory-like behaviors induced by ACPA

Two-way ANOVA and post-hoc analysis showed that pretest intra-BLA injection of sub-threshold dose of muscimol (0.025 µg/ mouse) or bicuculline (0.025 µg/mouse) potentiated %OAT (Figure 2(Fig. 2) Panel 4A for muscimol and Figure 2(Fig. 2) Panel 6A for bicuculline) and %OAE (Figure 2(Fig. 2) Panel 4B for muscimol and Figure 2(Fig. 2) Panel 6B for bicuculline) induced by ACPA in the retest day. The interventions showed that muscimol and bicuculline did not %OAT (Figure 2(Fig. 2) Panel 3A for muscimol and Figure 2(Fig. 2) Panel 5A for bicuculline) and %OAE (Figure 2(Fig. 2) Panel 3B for muscimol and Figure 2(Fig. 2) Panel 5B for bicuculline) induced by ACPA in the test day. Interesting data showed co-administration of muscimol or bicuculline with ACPA decreesed and increased respectively, locomotor activity both test and retest days (Figure 2(Fig. 2) Panel 3C and 4C for muscimol and Figure 2(Fig. 2) Panel 5C and 6C for bicuculline). Finally, the data revealed that the main effect of muscimol and bicuculline is on ACPA-induced locomotor activity rather than anxiety and aversive memory. All the experimental repeated measure results are summarized in the Table 2(Tab. 2).

## Discussion

It has been presumed that in the events which are connected to the feelings and emotions, the amygdala (a major brain region) regulates hippocampal formation activity for driving information recording to cortical areas. For instance, BLA activation into inactivation situation (i.e. damage) change memory from the event in favor of the essential part to visual details (Adolphs et al., 2001[2], 2005[3]; Canli et al., 2000[12]), thus, it seems that amygdala and the hippocampal system jointly play a critical role in the emotional memory improvement (Dolcos et al., 2004[19]). Moreover, emotional situation including aversion and fear, may improve or may weaken memory formation (McGaugh, 2004[46]). Since the existing animal models of learning and memory have a limited ability to detect the effect of drugs on anxiety and fear memory, as measured by these models, the exact subject matter and points may be misinterpreted and misunderstood. Therefore, elevated plus maze (EPM) task is an attempt to assess the effects of drugs on anxiety, learning, and memory happening at the same time in rodents (Asth et al., 2012[4]). The justification for utilizing the EPM in testing anxiety relies on the natural tendency of animals to avoid dangerous situation when they face height and open spaces (Chegini et al., 2014[15]; Zarrindast et al., 2010[82]).

### Effect of ACPA on open-arm exploratory behaviors in native mice subjected to the EPM 

The present results show that, intra-peritoneal infusion of selective CB1 cannabinoid receptor agonist, ACPA, make anxiolytic-like behaviors appear in the EPM. Moreover, anxiolytic effects of ACPA revealed in retest day. These results propose an impairment of aversive memory acquisition on testing presented itself in ACPA-treated groups. Meanwhile, the drug did not alter locomotor activity in the test and retest days. It has been showed that cannabinoids have several effects on the cognitive and non-cognitive behaviors such as short-term memory deficit, mood alterations, increased body awareness, decreased attention, sleepiness and discoordination (Court, 1998[17]; Heishman et al., 1997[28]). In terms of anxiety-like behaviors, it appears that the effects of cannabinoid agonists on this phenomenon are complex and often contradictory and conflicting in both humans and animals. In the anxiety animal model these agents induced dose-dependent regulation which seems the animal is strongly affected by environmental context. For instance, low doses of nabilone (Onaivi et al., 1990[57]), CP55, 940 (Marco et al., 2004[43]) and Delta9-tetrahydrocannabinol (Berrendero and Maldonado, 2002[8]) as CB1 cannabinoid receptor agonists induced anxiolytic-like effects in the EPM and light-dark tasks. On the other hand, the CB1 knockout mice also showed an anxiogenic-like behavior in the EPM and social interaction task (Haller et al., 2002[25]; Uriguen et al., 2004[72]). A recent study reported that cannabinoid agonists at high and low doses induced opposite effects on cognitive behaviors (Moreira and Wotjak, 2010[49]). For instance, high and low doses of these compounds induced anxiogenic- and anxiolytic-like behaviors, respectively. The effects can be blocked by CB1 cannabinoid antagonists (Haller et al., 2007[26]). 

A large body of evidence shows that the endocannabinoidergic system plays a crucial role in physiological mechanisms of learning and memory (Lichtman et al., 2002[40]; Marsicano et al., 2002[44]). For example, systemic injections of Δ9-THC, anandamide or intra-hippocampal injections of WIN55212-2 impair memory acquisition, consolidation, and recall in rodents (Costanzi et al., 2004[16]; Mishima et al., 2001[48]; Nasehi et al., 2010[51]). In terms of emotional memory, several brain regions including hippocampus, amygdala, and cortex with high density of CB1 receptor expression have critical role in emotional behavior regulation (Viveros et al., 2005[75]). It seems that the endocannabinoidergic system plays a major role in aversive memory extinction (Marsicano et al., 2002[44]). CB1 cannabinoid receptors are one of the major receptors in the emotional learning and memory and its neural plasticity process (Laviolette and Grace, 2006[39]; Marsicano et al., 2002[44]) and they have high expression in BLA and medial prefrontal cortex (mPFC) (McDonald and Mascagni, 2001[45]). 

### Effect of BLA GABA_A _agents on open-arm exploratory behaviors

The results showed that intra-BLA infusion of muscimol and bicuculline did not alter anxiety-like behaviors. Interestedly, further analyses showed that muscimol impaired memory formation and locomotor activity. A large number of studies have reported that amygdale plays an important role in anxiety-like behaviors (Roozendaal et al., 2009[64]); specifically amygdale BLA nucleus (Wang et al., 2011[76]). The amygdale BLA and lateral nucleus receives GABAergic neuron input from the raphe nucleus (Smith and Porrino, 2008[69]). This system and its receptors are involved in the regulation of cognitive and non-cognitive behaviors including anxiety-like behaviors, emotional memory, locomotor activity, attention, biorhythms, food intake and body temperature (Abrams et al., 2005[1]; Bonn et al., 2013[9]; Holmes, 2008[30]; Kriegebaum et al., 2010[38]). GABA is the major inhibitory neurotransmitter in the mammals CNS, including the brain stem. The GABA induces most of its effects through activation of either GABA_A_ or GABA_B_ receptors. Synaptically released GABA activates postsynaptic GABA_B_ receptors, which increase the membrane permeability to chloride, evoking a hyper-polarizing inhibitory postsynaptic current (IPSC). Inside the synapse, the concentration of GABA to a relatively high level (milimolar range) is increased by synaptic release (Cathala et al., 2005[14]; Farrant and Nusser, 2005[22]). The short current which is named as “phasic” inhibition happens as a result of the synaptic release of GABA from presynaptic terminals. But new detailed examinations have revealed that GABA released from presynaptic terminals can escape, or spillover, from the synaptic cleft, or there may be a spillover of GABA from the synaptic cleft, leading to activations of the receptors either at the synapse or distant from it (Cathala et al., 2005[14]; Farrant and Nusser, 2005[22]). On the contrary our results two previous studies showed that GABA_A _receptor activation induced anxiolytic-like behaviors (Naseri et al., 2014[53]; Rezayat et al., 2005[63]), Many drugs such as benzodiazepines, barbiturates and alcohols seem to elicits their effects via GABA_A_ receptors, (Morrow, 1995[50]). GABA_A_ receptors are ligand-gated heterooligomeric complexes comprising distinct subunits. Pharmacological studies have highlighted the crucial role of the GABAergic system in the regulation of anxiety. For instance, using pentylenetetrazole (a GABA_A_ receptors blocking agent) is shown to induce anxiety-like effects. On the contrary, using benzodiazepines (with increasing effect on GABAergic transmission) induce anxiolytic-like effect (Kalueff and Nutt, 1997[32]). The data from bicuculline-included experiments may suggest that under normal conditions, the BLA GABA_A_ receptors are not necessary for the anxiety-like behaviors and aversive memory formation. Some other studies have reported similar effects of intra-CA1 bicuculline on memory retention (Chegini et al., 2014[15]; Zarrindast et al., 2002[82]), spatial change and non-spatial novelty detection (Yousefi et al., 2013[81]).

### Effect of BLA GABAergic system on open-arm exploratory behavior induced by ACPA

The data uncovered that muscimol and bicuculline did not alter anxiolytic-like behaviors induced by ACPA, while both drugs restored ACPA-induced amnesia. Interestingly muscimol or bicuculline increased and decreased ACPA-induced locomotor activity, respectively. This mainly showed that there is a dealing mechanism between anxiety and cannabinoid level, to the extent that anxiety events increased endocannabinoid tone level for the reduction of anxiety phenomenon, for instance, the increase of anandamide following a foot shock after hearing in the amygdale (Marsicano et al., 2002[44]). Moreover, the contribution of amygdale endocannabinoids in the extinction of aversive memories has been also proposed (Azad et al., 2004[6]). It has been reported that in stressful stimuli, as well as rewarding experiences, mediate changes in the expression level of the CB1 receptor specifically in GABAergic terminals (Rossi et al., 2008[65]; Yamodo et al., 2010[80]). It is well worth considering that, the stress-mediated regulation of the GABAergic CB1 receptor has been postulated as a compensatory mechanism needed to restore the balance between GABAergic and glutamatergic neurotransmission in emotional homeostasis (Ruehle et al., 2012[66]). CB1 cannabinoid receptors are expressed in GABAergic terminals of the amygdale (Haring et al., 2007[27]). Thus, the endocannabinoidergic system can modulate GABAergic transmission through regulating the activity of afferents into GABA producing neurons (Haj-Dahmane and Shen, 2005[24]), and through directly modulating the functions of a subset of GABAergic neurons (Haring et al., 2007[27]). In the connection of cannabinoidergic and GABAergic systems interactions, some reports have mentioned that cannabinoids and their receptor agonists such as anandamide and ACPA inhibit the uptake of GABA into the cortical synaptosomes and this may happen through reducing the activity of the uptake energy source Na+/K+-ATPase (Steffens and Feuerstein, 2004[70]). Thus, using of cannabinoid receptor agonist, blocks respective transporters and finally increases GABA level in different brain regions (Köfalvi, 2007[36]). In vivo study showed GABA_A_ receptors have a critical role in modulation effect of cannabinoid (Beinfeld and Connolly, 2001[7]). For example it showed that blocked of GABA_A_ receptors by bicuculline completely restored Δ9-THC-induced deficits in both the Morris water maze working-memory task and an alternation T-maze task (Varvel et al., 2005[74]). However a study showed that microinjection of the GABA_A_ agonist muscimol into the central nucleus of the amygdala but not into the BLA nucleus of the amygdala, reduced the antinociceptive effects of systemic WIN55, 212-2 (Manning et al., 2003[42]), Rea and et al. (2013[62]) demonstrated that CB1 receptors in the BLA facilitate the expression of fear-conditioned analgesia, through a mechanism which is likely to involve the modulation of GABA_A_ signaling.

## Conclusion

In conclusion, the findings of the present study proposed that ACPA induced anxiolytic-like effect and aversive memory deficit. Furthermore, muscimol and bicuculline did not and restored anxiolytic-like effect and aversive memory deficit by ACPA, respectively. Moreover, muscimol or bicuculline increased and decreased locomotion by ACPA, respectively. It seems that the main effect of GABA_A_ in ACPA-induced behaviors is on locomotor activity rather than anxiety and aversive memory behaviors. 

## Acknowledgements

The authors thank the Iran National Science Foundation (INSF) for providing the financial support for the project.

## Conflicts of interest

There are no conflicts of interest.

## Figures and Tables

**Table 1:**
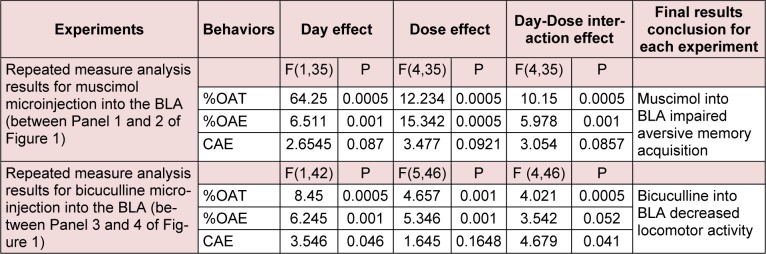
The table describes repeated measure analysis with P values for the effect of GABA_A _agonist and antagonist on exploratory-like behaviors.

**Table 2:**
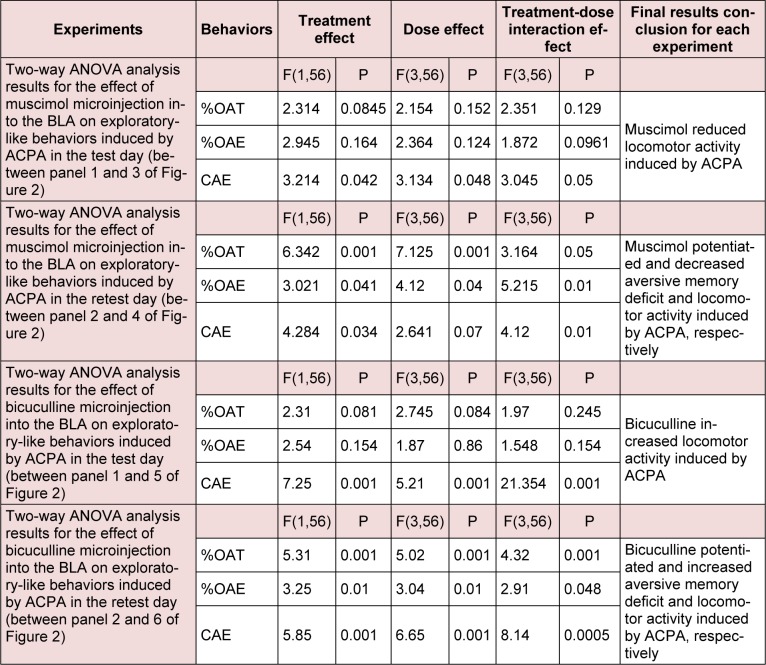
Repeated measure analysis with P values for the effect of ACPA by itself and two-way ANOVA results for the effect of GABA_A_ agonist and antagonist on exploratory-like behaviors induced by ACPA.
